# Echocardiographic Measurements in a Preclinical Model of Chronic Chagasic Cardiomyopathy in Dogs: Validation and Reproducibility

**DOI:** 10.3389/fcimb.2019.00332

**Published:** 2019-09-24

**Authors:** Eduardo B. Carvalho, Isalira P. R. Ramos, Alvaro F. S. Nascimento, Guilherme V. Brasil, Debora B. Mello, Martin Oti, Michael Sammeth, Maria T. Bahia, Antonio C. Campos de Carvalho, Adriana B. Carvalho

**Affiliations:** ^1^Carlos Chagas Filho Institute of Biophysics, Federal University of Rio de Janeiro, Rio de Janeiro, Brazil; ^2^National Center for Structural Biology and Bioimaging, Federal University of Rio de Janeiro, Rio de Janeiro, Brazil; ^3^School of Medicine, Federal University of Ouro Preto, Ouro Preto, Brazil; ^4^National Institute for Science and Technology in Regenerative Medicine, Federal University of Rio de Janeiro, Rio de Janeiro, Brazil

**Keywords:** Chagas disease, chagasic cardiomyopathy, canine model, dogs, systolic dysfunction

## Abstract

**Background:** The failure to translate preclinical results to the clinical setting is the rule, not the exception. One reason that is frequently overlooked is whether the animal model reproduces distinctive features of human disease. Another is the reproducibility of the method used to measure treatment effects in preclinical studies. Left ventricular (LV) function improvement is the most common endpoint in preclinical cardiovascular disease studies, while echocardiography is the most frequently used method to evaluate LV function. In this work, we conducted a robust echocardiographic evaluation of LV size and function in dogs chronically infected by *Trypanosoma cruzi*.

**Methods and Results:** Echocardiography was performed blindly by two distinct observers in mongrel dogs before and between 6 and 9 months post infection. Parameters analyzed included end-systolic volume (ESV), end-diastolic volume (EDV), ejection fraction (EF), and fractional shortening (FS). We observed a significant LVEF and FS reduction in infected animals compared to controls, with no significant variation in volumes. However, the effect of chronic infection in systolic function was quite variable, with EF ranging from 17 to 66%. Using the cut-off value of EF ≤ 40%, established for dilated cardiomyopathy (DCM) in dogs, only 28% of the infected dogs were affected by the chronic infection.

**Conclusions:** The canine model of CCC mimics human disease, reproducing the percentage of individuals that develop heart failure during the chronic infection. It is thus mandatory to establish inclusion criteria in the experimental design of canine preclinical studies to account for the variable effect that chronic infection has on systolic function.

## Introduction

Chagas disease is caused by the protozoan parasite *Trypanosoma cruzi*. This parasite can be transmitted to humans, domestic, or wild mammals and the infection is very difficult to cure. It is endemic in Latin America and has been spreading to non-endemic regions due to the migration of infected individuals. The estimated number of infected immigrants is over 300,000 in the United States and 80,000 in Europe (Rassi et al., 2010; Steverding, 2014). The classic mode of transmission involves *Triatomine* insects known as kissing bugs, but several other modes exist, such as blood transfusions, congenital, solid organ or bone marrow transplantation, and food-borne. After an acute phase characterized by non-specific symptoms, patients enter the chronic phase and can remain asymptomatic for the rest of their lives. However, ~30% of infected individuals will develop chronic chagasic cardiomyopathy (CCC), which is characterized by episodes of sudden cardiac death (SCD) due to complex ventricular arrhythmias, cardiac dilation and heart failure (HF). Survival is highly dependent on New York Heart Association (NYHA) functional class and on the presence of systolic dysfunction: 5-year mortality is over 80% in class III–IV patients (Rassi et al., 2006). The reason why only a subset of patients develop CCC remains unknown.

Current treatment recommendations for HF due to Chagas disease are identical to the ones given for other causes of HF (Rassi et al., 2006; de Andrade et al., 2011). Cardiac transplantation is further complicated in these patients by the possibility of parasite reactivation caused by immunosuppression. Large clinical trials have been conducted to investigate the efficacy of antiparasitic drugs (Rassi et al., 2006; de Andrade et al., 2011; Morillo et al., 2015) and cell therapy (dos Santos et al., 2012) for the treatment of CCC. Despite the evidence of beneficial effects available from small animal models (Garcia et al., 2005; Guarita-Souza, 2006; Goldenberg et al., 2008; Soares et al., 2011), both treatments were ineffective in human trials. The failure to translate preclinical results to the clinical setting is not new and the reasons are many, ranging from poor training in experimental design to the difficulty in publishing negative data (Collins and Tabak, 2014). However, one reason that is frequently overlooked is whether the animal model reproduces distinctive clinical and pathological features of human disease (Houser et al., 2012). Unfortunately, this is a major issue in rodent models of Chagas disease. Even though mice and rats usually respond to *T. cruzi* infection with cardiac inflammation and fibrosis, which are important hallmarks of CCC, they do not develop left ventricular dysfunction (Goldenberg et al., 2008; Soares et al., 2011; Jasmin et al., 2014; Mello et al., 2015). On the other hand, improvement of left ventricular function is commonly used as the primary endpoint in clinical trials of cardiac diseases. Therefore, in order to test the efficacy of alternative therapies for CCC, we need to find an animal model that reproduces this critical feature of human disease.

Dogs chronically infected by *T. cruzi* present cardiac inflammation, fibrosis, electrocardiographic alterations, and autoantibody production (de Lana et al., 1992; Guedes et al., 2009; Caldas et al., 2013; Daliry et al., 2014), which are all important characteristics of human CCC. However, data on left ventricular dysfunction are not robust. The literature reports a total of 16 dogs that were chronically infected by *T. cruzi* and had their cardiac function evaluated by echocardiography (Pascon et al., 2010; Sousa et al., 2011; Santos et al., 2012). The number of animals is too small to draw conclusions on this model's potential to reproduce human systolic dysfunction, a matter further aggravated by the large variance in ejection fraction (EF) reported in these publications. Nonetheless, the canine model is promising because dogs can naturally develop a form of dilated cardiomyopathy (DCM) that bears resemblance with human disease, including severe systolic dysfunction (Dukes-McEwan et al., 2003; Wess et al., 2017). We hypothesize the same could be true for CCC. Therefore, the objective of this work was to conduct a robust echocardiographic evaluation of left ventricular size and function in dogs chronically infected by *T. cruzi*.

## Materials and Methods

A more detailed version of these methods can be found in the Supplementary Material.

### Animals and Infection

Four month-old mongrel dogs (*n* = 78) were submitted to intraperitoneal inoculation of the VL-10 strain of *Trypanosoma cruzi* (discrete typing unit TcII isolated from human hosts in the State of Minas Gerais, Brazil) at a dose of 2,000 trypomastigotes/kg (Caldas et al., 2013, 2017). The VL-10 *T. cruzi* strain induces progressive heart fibrosis in dogs correlated with electrocardiographic alterations similar to those detected in humans (Caldas et al., 2013). Ten age-matched dogs were used as non-infected controls. Animals were fed commercial dog food and water *ad libitum*. All experiments were performed in conformity with the guidelines of the National Council for the Control of Animal Experimentation (Brazil) and the National Institutes of Health guide for the care and use of Laboratory animals (NIH Publications No. 8023, revised 1978). This study was approved by the local Committee on the Ethics of Animal Use of the Federal University of Ouro Preto under numbers 2012/18 and 2015/48.

Parasitemia was verified from the 10th day post infection by fresh blood examination in samples collected from the marginal ear vein. Additionally, anti-*T. cruzi* IgG antibodies were quantified in serum samples before, 30 and 180 days after *T. cruzi* infection by ELISA, as previously reported (Guedes et al., 2002).

### Echocardiography

Images were acquired before infection and between 6 and 9 months post infection. Dogs were anesthetized with intravenous sodium thiopental (15 mg/kg) and the precordial area was shaved. Data were collected with Esaote MyLab™30 Gold Cardiovascular or GE Healthcare Vivid i echocardiography equipment and analyzed with EchoPAC PC version 112 software. Images were measured independently by two blinded examiners. Left ventricular EF, end-systolic volume (ESV), and end-diastolic volume (EDV) were determined using the area-length (or bullet) method in two-dimensional mode images. Volumes were indexed by body surface area (BSA). Left ventricular fractional shortening (FS) was also determined in two-dimensional images.

### Statistics

Statistical analyses were conducted using R version 3.5.2 (R Core Team, 2017) with RStudio version 1.1.463 (RStudio Team, 2016) as a visual interface. Raw data, R packages and code used for analyses are provided in Supplementary Material. Correlation and level of agreement between echocardiographic measurements were determined by intraclass correlation coefficients (ICC) and Bland-Altman analysis (Giavarina, 2015), respectively. Interobserver reproducibility was determined by calculating percent differences between examiners (absolute difference between 2 measurements from the same image, divided by the mean of the 2 measurements). Volumes and systolic function were compared using Two-way ANOVA followed by Tukey's post-test to correct for multiple comparisons. Data were considered statistically significant if *p*-value was below 0.05.

## Results and Discussion

### Mortality of Experimental *T. cruzi* Infection in Dogs

All infected animals had trypomastigotes upon fresh blood examination between 10 and 20 days after inoculation. Accordingly, *T. cruzi*-specific IgG antibodies were detected in serum samples obtained from all infected dogs, remaining at high levels during the follow-up period (Supplementary Figure 1a).

Out of 78 dogs initially infected for this study, 25 animals died during the acute phase of infection, resulting in a mortality rate of 32%. The time period in which the animal deaths occurred varied from 1 to 6 months post infection (median 3, 1st Q 2.5, 3rd Q 5.5). Seven animals were excluded: 4 due to aggressive behavior, 2 in which the “post infection” echocardiographic exam was not conducted, and 1 was found to be pregnant after the study was initiated. The remaining 46 infected animals were used in this study (Supplementary Figure 1b). The observed mortality rate was much higher than expected for the acute phase of Chagas disease transmitted by insect vectors in humans, in which 0.25% of infected individuals die of severe myocarditis or meningoencephalitis (Rassi et al., 2010). However, mortality is dependent on transmission routes. For instance, food-borne transmission is reported to be more severe and to have mortality rates up to 35% in humans (Rassi et al., 2010). This is also the case in rodent animal models (Barreto-de-Albuquerque et al., 2015). In addition, mortality correlates to the dose of trypomastigotes administered at infection (Borges et al., 2013) and VL-10 strain is known to be aggressive and unresponsive to antiparasitic drugs even during the acute phase (Caldas et al., 2008, 2017), further contributing to the mortality observed in our dogs. The reason for using such an aggressive strain was to maximize cardiac tissue damage, as previous work by our group has shown that a smaller inoculum (<2,000 trypomastigotes/kg) or other inoculation routes would result in less tissue damage (Bahia et al., 2002).

### Alterations of Left Ventricular Size and Function in Canine Chronic Chagasic Cardiomyopathy

Our approach to minimize echocardiographic variability was to average the data analyzed by both examiners before comparing non-infected controls to *T. cruzi*-infected animals. Descriptive statistics are shown in Table 1.

**Table 1:** Descriptive statistics for volumes and systolic function.

**Parameter**	**Group**	**Time point**	**Min**	**1st Q**	**Median**	**Mean**	**3rd Q**	**Max**
EDV^*^	NI	BI	29.02	37.28	37.44	36.32	38.57	39.32
		PI	45.89	65.86	78.69	78.21	69.13	103.39
	Inf	BI	20.95	32.51	38.45	39.50	45.91	63.64
		PI	28.57	48.47	61.45	67.44	80.57	143.72
ESV^*^	NI	BI	9.07	10.47	10.96	12.23	11.83	18.83
		PI	20.42	27.83	35.41	34.95	39.93	55.30
	Inf	BI	7.04	11.10	14.09	16.35	18.85	38.53
		PI	13.20	25.99	33.64	37.56	42.76	119.17
EF	NI	BI	49.88	59.67	72.14	66.22	73.19	76.22
		PI	44.88	49.22	57.94	55.30	60.28	64.70
	Inf	BI	29.07	52.52	61.05	58.96	68.28	76.27
		PI	17.14	37.71	45.48	45.11	53.12	65.95
FS	NI	BI	0.291	0.295	0.314	0.365	0.433	0.493
		PI	0.202	0.238	0.273	0.277	0.300	0.405
	Inf	BI	0.051	0.186	0.259	0.277	0.384	0.488
		PI	0.031	0.152	0.192	0.202	0.256	0.449

* *Corrected for body surface area. PI, post infection; BI, before infection; Inf, infected; NI, non-infected*.

**Figure 1:**
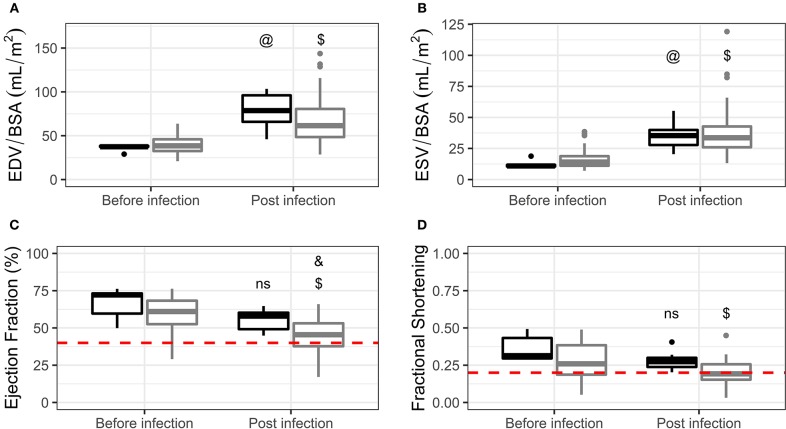
Volumes indexed by body surface area **(A,B)** and systolic function **(C,D)** comparisons in non-infected controls (black) and infected dogs (gray) before and post infection. Data are represented using Tukey's boxplots. @*p* < 0.05 compared to non-infected controls in the “before infection” time point. $*p* < 0.05 compared to infected dogs in the “before infection” time point. &*p* < 0.05 compared to non-infected controls in the “post infection” time point. ns, not significant compared to non-infected controls in the “before infection” time point. Red dashed lines represent proposed inclusion criteria cut-offs (see “Establishment of inclusion criteria” in the main text).

No differences were found between experimental groups in the before infection time point in EDV, ESV, EF, or FS (Figures 1A–D, respectively). Both groups, infected and non-infected, exhibited a significant increase in EDV and ESV when compared to baseline, even after indexing for BSA. No differences were present in EDV (Figure 1A) or ESV (Figure 1B) when comparing infected and non-infected dogs in the post infection time point. On the other hand, infected animals exhibited a significant decrease in EF when compared to baseline and to non-infected controls in the post infection time point (Figure 1C). Non-infected dogs did not show a significant decrease in EF when compared to baseline. FS was significantly decreased in infected dogs when compared to baseline, although no differences were present between this group and non-infected controls in the post infection time point (Figure 1D).

**Figure 2:**
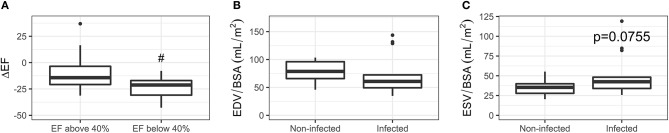
**(A)** After subsetting dogs using the 40% EF cut-off, ΔEF was calculated by subtracting post infection by before infection values for each infected animal. Dogs with EF below 40% had a significantly higher variation between time points (#*p* < 0.05). **(B,C)** Volumes indexed by body surface area of the subset of dogs that had EF below 40%. Data are represented using Tukey's boxplots.

Even though we observed an overall EF reduction in infected animals, it is important to note that EF values ranged from 17.14 to 65.95% (Table 1). Therefore, the effect of chronic *T. cruzi* infection in systolic function can be quite diverse in dogs, varying from normal to severe dysfunction. Although this may be interpreted as a weakness of the model, one needs to remember that the same is observed in humans. Some patients remain asymptomatic for the rest of their lives while, for unknown reasons, ~30% develop CCC. In this context, one of the proposed criteria for the diagnosis of canine idiopathic DCM uses the EF cut-off value of 40% (Dukes-McEwan et al., 2003). Applying this cut-off to our data, CCC would be present in 28.2% of the infected dogs, which is very similar to the proportion of patients that develop the disease. Importantly, this subset of dogs had a significantly higher reduction in EF (ΔEF = EF post infection – EF before infection) when compared to dogs that fall above the cut-off (Figure 2A). In addition, reference values (mean ± 2 SD) for our 10 age-matched non-infected dogs range from 41.01 to 69.57%, further supporting the 40% EF cut-off. Finally, this number falls well within the established criteria for cardiac dysfunction in humans (Lang et al., 2015) and children (Lopez et al., 2010).

FS has a much more robust dataset available for dogs in the literature. A meta-analysis of 1,152 normal adult animals (including 22 different breeds and mongrels) reports FS of 0.34 ± 0.051 (Hall et al., 2008). Assuming a normal distribution, reference values (mean ± 2 SD) would range from 0.238 to 0.442. Our subset of dogs with EF below 40% also falls below the FS 0.238 cut-off, with values ranging from 0.031 to 0.194.

Cardiac dilation is another important aspect of CCC. We observed an increase in EDV comparing 4 month-old (before infection) to 10–13 month-old (post infection) dogs, even after indexing for BSA. Since, this is observed in both non-infected and infected animals, we believe it is due to a larger physiological growth rate of the heart in relation to the body in pups transitioning to adulthood (Northup et al., 1957). Moreover, volumes can be lower in pups due to the higher heart rates at this age (Bayón et al., 1994). Therefore, comparisons between non-infected controls and infected dogs in the post infection time point (when all dogs are adults) are more valuable to evaluate cardiac dilation in our model.

Chronic infection did not lead to overall cardiac dilation in adult dogs (Figure 1A). One could expect that our animals with EF below 40% would have higher EDV and/or ESV values. However, there were no differences between this subset of infected dogs in comparison to non-infected controls (Figures 2B,C), although we did observe a borderline *p*-value for ESV (0.0755). Severe cardiomegaly and systolic dysfunction tend to co-exist in stage 4 chagasic cardiomyopathy patients (Rassi et al., 2010). Nevertheless, this does not happen in earlier stages, especially stage 3, in which patients can present diffuse LV wall motion abnormalities but only mild to moderate cardiomegaly (Rassi et al., 2010). In fact, cardiac dilation and systolic dysfunction are considered independent risk factors in the prognostic evaluation of CCC (Rassi et al., 2006), underscoring the heterogeneous presentation of the disease. Thus, the presence of systolic dysfunction but not overt cardiac dilation in our model is compatible with the clinical characteristics described for stage 3 CCC.

### Reproducibility of Echocardiography in Canine Chronic Chagasic Cardiomyopathy

LV function improvement is the most common endpoint in cardiovascular disease studies, while echocardiography is the most frequently used method to evaluate LV function (Lang et al., 2015). Therefore, reproducibility is crucial not only in clinical trials but also in preclinical studies to increase the likelihood that treatment effects are translatable to the clinical setting. Unfortunately, this type of analysis is rare in preclinical models and there are no studies in the literature investigating echocardiographic reproducibility in dogs. For this reason, we compared our data to the human pediatric population, which is more similar to dogs at least in weight, size, and heart rate. Moreover, our dogs were infected at a young age, thus, the effects of physiological growth must be taken into consideration, as in children.

Echocardiographic images were independently measured by two blinded examiners. ICC and Bland-Altman analyses for long axis length (Supplementary Figures 2a–d), short axis area (Supplementary Figures 2e–h), and short axis length (Supplementary Figures 2i–l) in systole and diastole were conducted. We observed a high correlation (0.85 < ICC <0.97, Supplementary Table 1 and Supplementary Figure 3a) between examiners for all measurements. Bland-Altman plots show an average difference between examiners below 0.21 mm for length and 0.45 mm^2^ for area (Supplementary Table 1 and Supplementary Figure 3b).

The ventricular volume variability (VVV) study from the Pediatric Heart Network reported mean percent differences between examiners for length measurements between 5 and 7% (Colan et al., 2012). In our case, this range was from 6 to 11% (Supplementary Table 1 and Supplementary Figure 3c). Anatomic differences between dogs and humans might account for this small increase in variability: the larger antero-posterior thoracic diameter in dogs makes it more challenging to obtain high quality echocardiographic images. Area measurements had mean percent differences between 11 and 19% (Supplementary Table 1 and Supplementary Figure 3c), higher than the differences in length. This was also observed in the pediatric population (Selamet Tierney et al., 2017).

**Figure 3:**
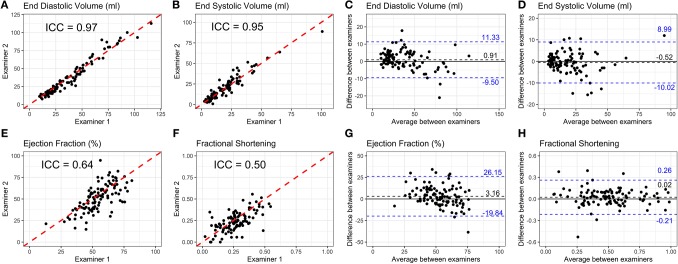
**(A,B,E,F)** Scatterplots and intraclass correlation coefficients (ICC) for left ventricular end diastolic volume, end systolic volume, ejection fraction, and fractional shortening, calculated from the measurements obtained by 2 independent blinded examiners. The proximity of a data point to the dashed red line indicates a high degree of agreement between the measures of both examiners. **(C,D,G,H)** Bland-Altman plots showing differences between examiners on the Y axis and averaged values for both examiners on the X axis for each measurement. The dashed black line indicates the mean difference between examiners considering all measurements and the dashed blue lines represent 95% limits of agreement between examiners.

ICC and Bland-Altman plots for volume extrapolations using the area-length method are illustrated in Figure 3. This method was chosen because it has been demonstrated to have higher reproducibility in children (Margossian et al., 2015). There was a high correlation and low mean difference between examiners for EDV (ICC = 0.97, difference = 0.93 mL, Figures 3A,C) and ESV (ICC = 0.95, difference = −0.49 mL, Figures 3B,D). EF had lower correlation between examiners (ICC = 0.64, Figure 3E), while the mean difference was higher (3.16, Figure 3G). Variability between examiners in ejection fraction estimations is a known issue in echocardiography (Cantinotti and Koestenberger, 2017). The VVV study reported that, together with slopes, variables that are extrapolated from direct measurements have the highest percent differences among 119 echocardiography variables examined (Colan et al., 2012). If calculated from 2 measures, as is the case for volumes, the interobserver mean percent difference was 13.6%. If calculated from 4 measures, the case of ejection fraction, the interobserver mean percent difference was 22.2%. In another study, the Pediatric Heart Network reported mean percent differences between examiners for EDV and EF to be 10.3 and 12.8%, respectively (Selamet Tierney et al., 2017). Data for ESV were not available. In our study, mean percent differences for EDV and EF were 12.79 and 18.47%, respectively (Supplementary Table 1 and Supplementary Figure 3c), within the range of values reported for the pediatric population.

FS had lower correlation (ICC = 0.50, Figure 3F) and higher mean percent difference (26.3%, Supplementary Table 1 and Supplementary Figure 3c) than EF, but we kept this parameter in our study because it is the most frequently used for the evaluation of cardiac function in dogs.

### The Need for Establishment of Inclusion Criteria

Given the results reported above, we consider that our canine model of CCC is a viable option for preclinical testing of new therapies. However, if the endpoint involves improvement of systolic function, inclusion criteria are mandatory. We propose that the cut-off value of 40% EF be used to include animals in treatment groups (red dashed line in Figure 1C). This cut-off value is also a major criteria for the diagnosis of idiopathic DCM proposed by the European Society of Veterinary Cardiology (Dukes-McEwan et al., 2003). Dogs with EF between 40 and 50% might be used as a separate group to evaluate treatment effects in a mild systolic dysfunction scenario. The use of EF is recommended over FS due to the lower interobserver variability. If only FS is available, the 0.238 value might be adopted based on the robust metanalysis published by Hall (Hall et al., 2008). But we advise caution in this case since such a cut-off would lead to the inclusion of 63% of our infected animals. FS values below 0.25 may be considered abnormally low. However, this is highly breed specific. We suggest the more stringent value of 0.20 (red dashed line in Figure 1D), in agreement with the value recommended by the canine idiopathic DCM guidelines (Dukes-McEwan et al., 2003). The use of EF is also recommended over volumes, especially if dogs are to be infected at a young age.

### Limitations

The inclusion criteria proposed create operational concerns for the model. If only ~30% of infected animals are included, there will be considerable increases in cost and space requirements to reach a sufficient number of animals for preclinical testing, but studies will gain translational robustness. Echocardiography interobserver variability, another limitation, could still be reduced using multibeat averages as opposed to single beat measurements (Colan et al., 2012). Reducing variability can impact sample size requirements and improve the cost and space concerns raised above. Finally, we cannot rule out that longer periods of observation of chronic infection could increase the proportion of dogs with systolic dysfunction. Further studies need to be conducted to address this possibility.

## Conclusions

The dog model of CCC reproduces most of the features of the human disease, including the low percentage of animals (~30%) that develop LV dysfunction post infection. But, if therapies are to be tested in this model, and improvement in LVEF is the target endpoint, inclusion criteria are mandatory and LVEF by echocardiography must be below 40%.

## Data Availability Statement

The datasets generated for this study can be found in the Supplementary Material.

## Ethics Statement

The animal study was reviewed and approved by Committee on the Ethics of Animal Use of the Federal University of Ouro Preto.

## Author Contributions

EC and IR conducted echocardiography image acquisition and analysis. AN conducted animal infection and helped with echocardiography image acquisition. GB conducted echocardiography image acquisition. DM conducted data analysis and helped with echocardiography image acquisition. MO and MS conducted data analysis. MB participated in the conception and design, and provided funding for the study. ACC participated in the conception and design, provided funding for the study, and wrote the manuscript. ABC participated in the conception, design and data analysis, provided funding for the study, and wrote the manuscript.

### Conflict of Interest

The authors declare that the research was conducted in the absence of any commercial or financial relationships that could be construed as a potential conflict of interest.
