# Supplementary material for: Echocardiographic Measurements in a Preclinical Model of Chronic Chagasic Cardiomyopathy in Dogs: Validation and Reproducibility
Source: Front Cell Infect Microbiol. 2019 Sep 24;9:332. doi: 10.3389/fcimb.2019.00332 (PMC6768978; doi:10.3389/fcimb.2019.00332)
Supplement: Supplementary file 1 [file Table_1.pdf]

Supplementary Material

1 Supplementary Methods

Echocardiography

Image acquisition

All sonographers were trained to operate Esaote MyLab™30 Gold Cardiovascular or GE Healthcare Vivid i equipments. Image acquisition was conducted by 3 different sonographers. The exams each sonographer conducted were not pre-defined because it involved traveling to another state, where the dogs were housed, and was dependent on each sonographer’s schedule. Dogs were anesthetized by intravenous injection of 15 mg/kg of sodium thiopental through the cephalic vein and positioned in right lateral decubitus. The precordial area was shaved and electrocardiography electrodes were positioned on arms and legs. A conductive gel was placed between the skin and a 2.9-5.7 MHz multifrequency transducer. Two-dimensional cineloops of the left ventricle were recorded in the apical four chamber and parasternal short axis views.

Image analysis

All exams were coded by a person not involved in image acquisition or analysis. Each exam received a random number to ensure blindness. After the images were coded, they were sent to 2 examiners, who also participated in image acquisition. Together, they conducted a quality assessment to determine if the image was usable. If the structure was not properly recorded or was considered too poorly defined to be measured, it was excluded. This happened to 3 out of 107 exams. Then, examiners chose the best cineloop out of all that were available and, together, decided which frame should be measured in systole and diastole. The systolic frame was chosen using the moment right before mitral valve opening as a guide (or the image in which the ventricle was smallest). The diastolic frame was chosen using the moment right before mitral valve closure (or the first frame within the QRS complex). After agreeing on quality assessment and frames, examiners measured each image independently on a later date. The following measurements were obtained: long axis length in systole and diastole (apical four chamber view), short axis area in systole and diastole and short axis length in systole and diastole (parasternal short axis view). Choice of the exact position to measure length was always determined to be the largest possible length in the image. End diastolic and systolic volumes were calculated using the area-length (or bullet) method, according to the following equation:

Volume (mL) = [5*(short axis area)*(long axis length)]/6

These volumes were then indexed by body surface area (BSA), estimated by the following equation:

BSA (m2) = 10.1*(weight in grams)0.667*10-4

Ejection fraction (EF) was calculated by:

EF (%) = (EDV – ESV)/EDV

Fractional shortening (FS) was calculated in parasternal short axis images by:

FS (%) = (length in diastole – length in systole)/length in diastole.

2 Supplementary Figures

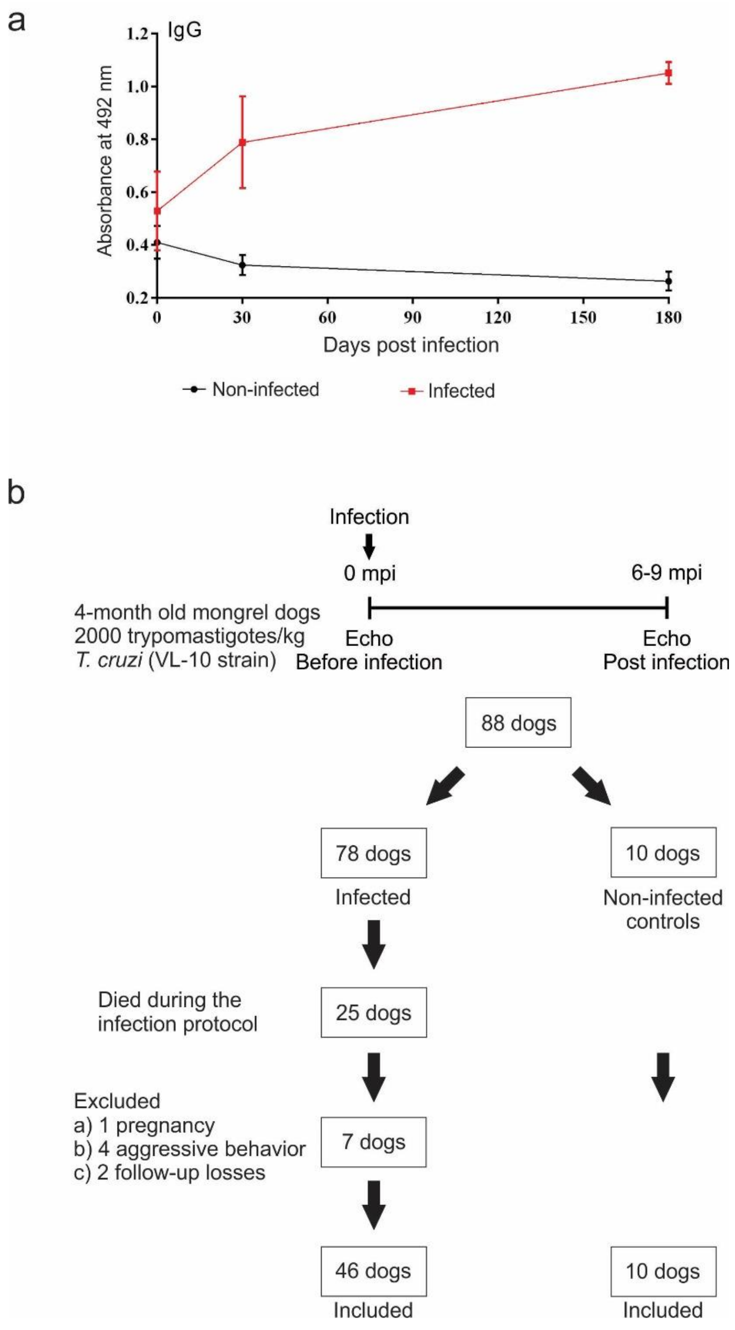

Supplementary Figure 1. (a) *T. cruzi*-specific IgG antibodies in sera samples collected before, 30 and 180 days after infection of dogs with VL-0 strain. (b) Study flow diagram showing the number of dogs included in the final analysis. mpi: months post infection.

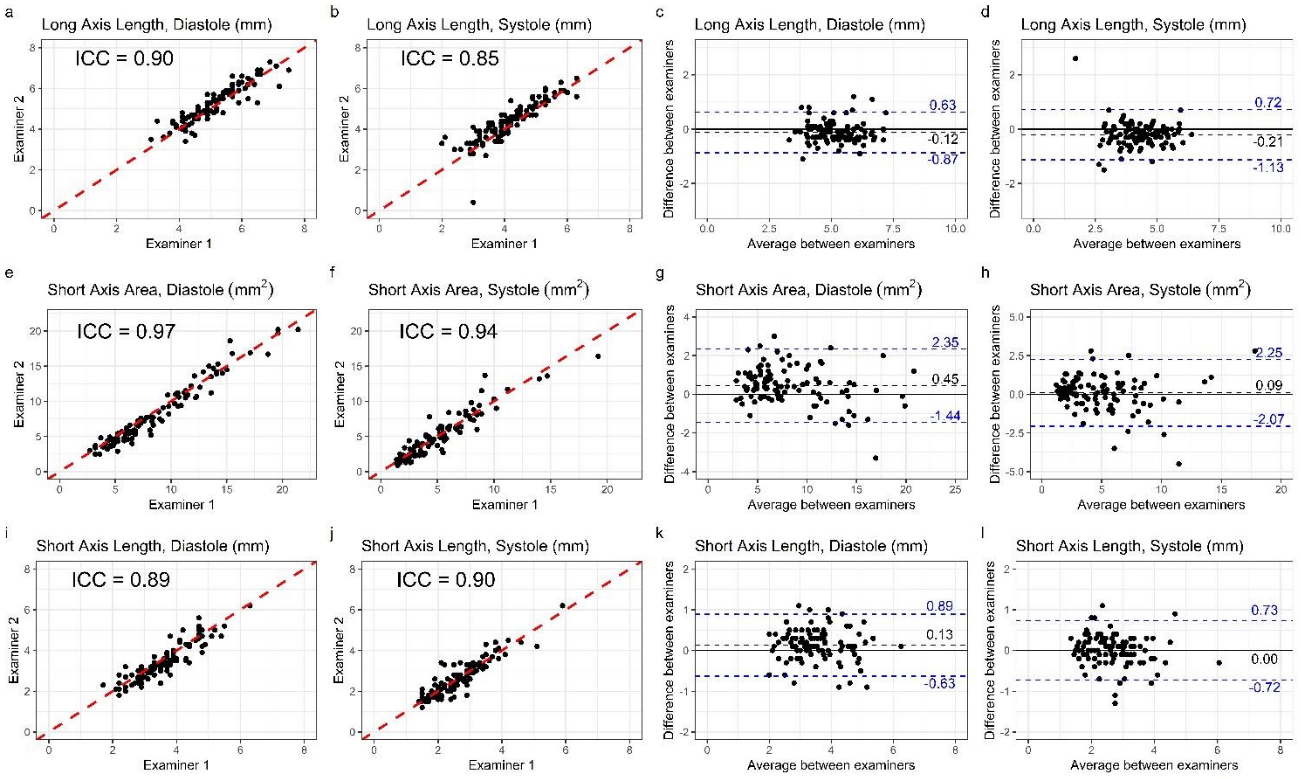

Supplementary Figure 2. (a, b, e, f, i, j) Scatterplots and intraclass correlation coefficients (ICC) for echocardiographic measurements collected by 2 independent blinded examiners. The proximity of a data point to the dashed red line indicates a high degree of agreement between the measures of both examiners. (c, d, g, h, k, l) Bland-Altman plots showing differences between examiners on the Y axis and averaged values for both examiners on the X axis for each measurement. The dashed black line indicates the mean difference between examiners considering all measurements and the dashed blue lines represent 95% limits of agreement between examiners.

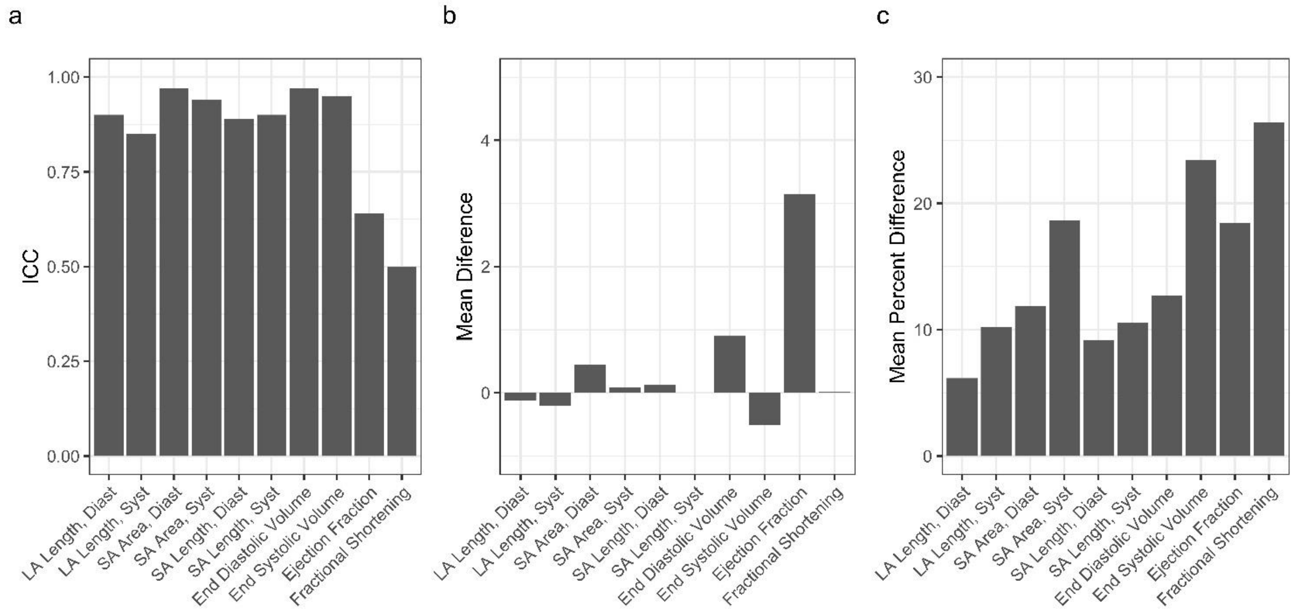

Supplementary Figure 3. Intraclass correlation coefficients (ICC), mean difference and mean percent differences between examiners for echocardiographic measurements. LA: long axis, SA: short axis, Diast: diastole, Syst: systole.

Supplementary Table 1: ICCs, mean differences, 95% limits of agreement and mean percent differences of echocardiography measurements between blinded examiners.

	Intraclass correlation coefficients	Mean difference	95% limits of agreement	Mean percent difference
Long axis length, diastole	0.90	-0.12	-0.87; 0.63	6.16
Long axis length, systole	0.85	-0.21	-1.13; 0.72	10.18
Short axis area, diastole	0.97	0.45	-1.44; 2.35	11.87
Short axis area, systole	0.94	0.09	-2.07; 2.25	18.68
Short axis length, diastole	0.89	0.13	-0.63; 0.89	9.20
Short axis length, systole	0.90	0.00	-0.72; 0.73	10.53
EDV	0.97	0.91	-9.50; 11.33	12.71
ESV	0.95	-0.51	-10.02; 8.99	23.43
EF	0.64	3.15	-19.84; 26.15	18.45
FS	0.50	0.02	-0.21; 0.26	26.42

Units: length – mm, area – mm^2, volume – ml
