# Supplementary material for: Echocardiographic Measurements in a Preclinical Model of Chronic Chagasic Cardiomyopathy in Dogs: Validation and Reproducibility
Source: Front Cell Infect Microbiol. 2019 Sep 24;9:332. doi: 10.3389/fcimb.2019.00332 (PMC6768978; doi:10.3389/fcimb.2019.00332)
Supplement: Supplementary file 2 [file Table_2.docx]

##################################

#Echocardiographic analyzes#

##################################

# Load R packages, filter and organize data

library(plyr)

library(dplyr)

library(tidyr)

library(ggplot2)

library(gridExtra)

library(car)

library(psych)

data <- read.csv('Raw data.csv')

data <- mutate(data,

twoD.FS = (Short.axis.diastole.largest.length - Short.axis.systole.largest.length)/Short.axis.diastole.largest.length)

data <- mutate(data, Weight.g = Weight*1000)

data <- mutate(data, BSA = 10.1*(Weight.g^0.667)*0.0001)

data <- mutate(data, Average.EDV.bsa = Average.EDV/BSA)

data <- mutate(data, Average.ESV.bsa = Average.ESV/BSA)

data <- transform(data, Timepoint = revalue(Timepoint, c('After infection' = 'Post infection')))

data$Timepoint <- ordered(data$Timepoint, levels = c('Before infection', 'Post infection'))

data$Treatment <- ordered(data$Treatment, levels = c('Non-infected', 'Infected'))

examiner1 <- filter(data, Examiner == 'Examiner 1')

examiner2 <- filter(data, Examiner == 'Examiner 2')

examiner1 <- mutate(examiner1, Average.twoD.FS = (examiner1$twoD.FS + examiner2$twoD.FS) / 2)

##### SUPPL FIGURE 2 #####

# Correlations between measurements

icc1 <- select(data, Examiner, Code, Long.axis.diastole.length) %>% na.omit()

icc1 <- spread(icc1, Examiner, Long.axis.diastole.length)

icc1.1 <- select(icc1, 'Examiner 1', 'Examiner 2')

cor1 <- ICC(icc1.1)

p1 <- ggplot(aes(examiner1$Long.axis.diastole.length, y = examiner2$Long.axis.diastole.length),

data = examiner1, examiner2) +

geom_point() +

xlim(0, 8) + ylim(0, 8) +

labs(title = 'Long Axis Length, Diastole (mm)', x = 'Examiner 1', y = 'Examiner 2', tag = 'a') +

theme(plot.title = element_text(size = 11)) + theme_bw() +

annotate('text', x = 2.25, y = 7.5, size = 6, label = 'ICC = 0.90') +

geom_abline(color = 'red', size = 1, linetype = 2)

icc2 <- select(data, Examiner, Code, Long.axis.systole.length) %>% na.omit()

icc2 <- spread(icc2, Examiner, Long.axis.systole.length)

icc2.1 <- select(icc2, 'Examiner 1', 'Examiner 2')

cor2 <- ICC(icc2.1)

p2 <- ggplot(aes(x = examiner1$Long.axis.systole.length, y = examiner2$Long.axis.systole.length),

data = examiner1, examiner2) +

geom_point() +

xlim(0, 8) + ylim(0, 8) +

labs(title = 'Long Axis Length, Systole (mm)', x = 'Examiner 1', y = 'Examiner 2', tag = 'b') +

theme(plot.title = element_text(size = 11)) + theme_bw() +

annotate('text', x = 2.25, y = 7.5, size = 6, label = 'ICC = 0.85') +

geom_abline(color = 'red', size = 1, linetype = 2)

icc3 <- select(data, Examiner, Code, Short.axis.diastole.area) %>% na.omit()

icc3 <- spread(icc3, Examiner, Short.axis.diastole.area)

icc3.1 <- select(icc3, 'Examiner 1', 'Examiner 2')

cor3 <- ICC(icc3.1)

p3 <- ggplot(aes(x = examiner1$Short.axis.diastole.area, y = examiner2$Short.axis.diastole.area),

data = examiner1, examiner2) +

geom_point() +

xlim(0, 22) + ylim(0, 22) +

labs(title = 'Short Axis Area, Diastole' ~(mm^2), x = 'Examiner 1', y = 'Examiner 2', tag = 'e') +

theme(plot.title = element_text(size = 11)) + theme_bw() +

annotate('text', x = 6, y = 20, size = 6, label = 'ICC = 0.97') +

geom_abline(color = 'red', size = 1, linetype = 2)

icc4 <- select(data, Examiner, Code, Short.axis.systole.area) %>% na.omit()

icc4 <- spread(icc4, Examiner, Short.axis.systole.area)

icc4.1 <- select(icc4, 'Examiner 1', 'Examiner 2')

cor4 <- ICC(icc4.1)

p4 <- ggplot(aes(x = examiner1$Short.axis.systole.area, y = examiner2$Short.axis.systole.area),

data = examiner1, examiner2) +

geom_point() +

xlim(0, 22) + ylim(0, 22) +

labs(title = 'Short Axis Area, Systole' ~(mm^2), x = 'Examiner 1', y = 'Examiner 2', tag = 'f') +

theme(plot.title = element_text(size = 11)) + theme_bw() +

annotate('text', x = 6, y = 20, size = 6, label = 'ICC = 0.94') +

geom_abline(color = 'red', size = 1, linetype = 2)

icc5 <- select(data, Examiner, Code, Short.axis.diastole.largest.length) %>% na.omit()

icc5 <- spread(icc5, Examiner, Short.axis.diastole.largest.length)

icc5.1 <- select(icc5, 'Examiner 1', 'Examiner 2')

cor5 <- ICC(icc5.1)

p5 <- ggplot(aes(x = examiner1$Short.axis.diastole.largest.length, y = examiner2$Short.axis.diastole.largest.length),

data = examiner1, examiner2) +

geom_point() +

xlim(0, 8) + ylim(0, 8) +

labs(title = 'Short Axis Length, Diastole (mm)', x = 'Examiner 1', y = 'Examiner 2', tag = 'i') +

theme(plot.title = element_text(size = 11)) + theme_bw() +

annotate('text', x = 2.25, y = 7.5, size = 6, label = 'ICC = 0.89') +

geom_abline(color = 'red', size = 1, linetype = 2)

icc6 <- select(data, Examiner, Code, Short.axis.systole.largest.length) %>% na.omit()

icc6 <- spread(icc6, Examiner, Short.axis.systole.largest.length)

icc6.1 <- select(icc6, 'Examiner 1', 'Examiner 2')

cor6 <- ICC(icc6.1)

p6 <- ggplot(aes(x = examiner1$Short.axis.systole.largest.length, y = examiner2$Short.axis.systole.largest.length),

data = examiner1, examiner2) +

geom_point() +

xlim(0, 8) + ylim(0, 8) +

labs(title = 'Short Axis Length, Systole (mm)', x = 'Examiner 1', y = 'Examiner 2', tag = 'j') +

theme(plot.title = element_text(size = 11)) + theme_bw() +

annotate('text', x = 2.25, y = 7.5, size = 6, label = 'ICC = 0.90') +

geom_abline(color = 'red', size = 1, linetype = 2)

# Bland-Altman plots for measurements

bland.altman <- mutate(examiner1, difference.LADL = examiner1$Long.axis.diastole.length - examiner2$Long.axis.diastole.length,

mean.LADL = (examiner1$Long.axis.diastole.length + examiner2$Long.axis.diastole.length) / 2,

difference.LASL = examiner1$Long.axis.systole.length - examiner2$Long.axis.systole.length,

mean.LASL = (examiner1$Long.axis.systole.length + examiner2$Long.axis.systole.length) / 2,

difference.SADA = examiner1$Short.axis.diastole.area - examiner2$Short.axis.diastole.area,

mean.SADA = (examiner1$Short.axis.diastole.area + examiner2$Short.axis.diastole.area) / 2,

difference.SASA = examiner1$Short.axis.systole.area - examiner2$Short.axis.systole.area,

mean.SASA = (examiner1$Short.axis.systole.area + examiner2$Short.axis.systole.area) / 2,

difference.SADL = examiner1$Short.axis.diastole.largest.length - examiner2$Short.axis.diastole.largest.length,

mean.SADL = (examiner1$Short.axis.diastole.largest.length + examiner2$Short.axis.diastole.largest.length) / 2,

difference.SASL = examiner1$Short.axis.systole.largest.length - examiner2$Short.axis.systole.largest.length,

mean.SASL = (examiner1$Short.axis.systole.largest.length + examiner2$Short.axis.systole.largest.length) / 2)

bland.altman <- mutate(bland.altman, percent.difference.LADL = difference.LADL/mean.LADL,

percent.difference.LASL = difference.LASL/mean.LASL,

percent.difference.SADL = difference.SADL/mean.SADL,

percent.difference.SASL = difference.SASL/mean.SASL,

percent.difference.SADA = difference.SADA/mean.SADA,

percent.difference.SASA = difference.SASA/mean.SASA)

mean(abs(bland.altman$percent.difference.LADL), na.rm = TRUE)

mean(abs(bland.altman$percent.difference.LASL), na.rm = TRUE)

mean(abs(bland.altman$percent.difference.SADL), na.rm = TRUE)

mean(abs(bland.altman$percent.difference.SASL), na.rm = TRUE)

mean(abs(bland.altman$percent.difference.SADA), na.rm = TRUE)

mean(abs(bland.altman$percent.difference.SASA), na.rm = TRUE)

line.mean.p7 <- mean(bland.altman$difference.LADL, na.rm = TRUE)

line.upper.p7 <- mean(bland.altman$difference.LADL, na.rm = TRUE) + (sd(bland.altman$difference.LADL, na.rm = TRUE) * 1.96)

line.lower.p7 <- mean(bland.altman$difference.LADL, na.rm = TRUE) - (sd(bland.altman$difference.LADL, na.rm = TRUE) * 1.96)

p7 <- ggplot(aes(x = bland.altman$mean.LADL, y = bland.altman$difference.LADL),

data = bland.altman) +

geom_point() +

xlim(0, 10) + ylim(-3, 3) +

labs(title = 'Long Axis Length, Diastole (mm)', x = 'Average between examiners',

y = 'Difference between examiners', tag = 'c') +

theme(plot.title = element_text(size = 11)) + theme_bw() +

geom_hline(yintercept = 0, linetype = 1) +

geom_hline(yintercept = line.mean.p7, linetype = 2) +

geom_hline(yintercept = line.upper.p7, linetype = 2, color = 'blue') +

geom_hline(yintercept = line.lower.p7, linetype = 2, color = 'blue') +

annotate('text', x = 9.5, y = -0.35, size = 4, label = sprintf('%0.2f',line.mean.p7)) +

annotate('text', x = 9.5, y = 0.9, size = 4, color = 'blue', label = sprintf('%0.2f',line.upper.p7)) +

annotate('text', x = 9.5, y = -1.1, size = 4, color = 'blue', label = sprintf('%0.2f',line.lower.p7))

line.mean.p8 <- mean(bland.altman$difference.LASL, na.rm = TRUE)

line.upper.p8 <- mean(bland.altman$difference.LASL, na.rm = TRUE) + (sd(bland.altman$difference.LASL, na.rm = TRUE) * 1.96)

line.lower.p8 <- mean(bland.altman$difference.LASL, na.rm = TRUE) - (sd(bland.altman$difference.LASL, na.rm = TRUE) * 1.96)

p8 <- ggplot(aes(x = bland.altman$mean.LASL, y = bland.altman$difference.LASL),

data = bland.altman) +

geom_point() +

xlim(0, 10) + ylim(-3, 3) +

labs(title = 'Long Axis Length, Systole (mm)', x = 'Average between examiners',

y = 'Difference between examiners', tag = 'd') +

theme(plot.title = element_text(size = 11)) + theme_bw() +

geom_hline(yintercept = 0, linetype = 1) +

geom_hline(yintercept = line.mean.p8, linetype = 2) +

geom_hline(yintercept = line.upper.p8, linetype = 2, color = 'blue') +

geom_hline(yintercept = line.lower.p8, linetype = 2, color = 'blue') +

annotate('text', x = 9.5, y = -0.45, size = 4, label = sprintf('%0.2f',line.mean.p8)) +

annotate('text', x = 9.5, y = 1, size = 4, color = 'blue', label = sprintf('%0.2f',line.upper.p8)) +

annotate('text', x = 9.5, y = -1.4, size = 4, color = 'blue', label = sprintf('%0.2f',line.lower.p8))

line.mean.p9 <- mean(bland.altman$difference.SADL, na.rm = TRUE)

line.upper.p9 <- mean(bland.altman$difference.SADL, na.rm = TRUE) + (sd(bland.altman$difference.SADL, na.rm = TRUE) * 1.96)

line.lower.p9 <- mean(bland.altman$difference.SADL, na.rm = TRUE) - (sd(bland.altman$difference.SADL, na.rm = TRUE) * 1.96)

p9 <- ggplot(aes(x = bland.altman$mean.SADL, y = bland.altman$difference.SADL),

data = bland.altman) +

geom_point() +

xlim(0, 8) + ylim(-2, 2) +

labs(title = 'Short Axis Length, Diastole (mm)', x = 'Average between examiners',

y = 'Difference between examiners', tag = 'k') +

theme(plot.title = element_text(size = 11)) + theme_bw() +

geom_hline(yintercept = 0, linetype = 1) +

geom_hline(yintercept = line.mean.p9, linetype = 2) +

geom_hline(yintercept = line.upper.p9, linetype = 2, color = 'blue') +

geom_hline(yintercept = line.lower.p9, linetype = 2, color = 'blue') +

annotate('text', x = 7.5, y = 0.4, size = 4, label = sprintf('%0.2f',line.mean.p9)) +

annotate('text', x = 7.5, y = 1.1, size = 4, color = 'blue', label = sprintf('%0.2f',line.upper.p9)) +

annotate('text', x = 7.5, y = -0.8, size = 4, color = 'blue', label = sprintf('%0.2f',line.lower.p9))

line.mean.p10 <- mean(bland.altman$difference.SASL, na.rm = TRUE)

line.upper.p10 <- mean(bland.altman$difference.SASL, na.rm = TRUE) + (sd(bland.altman$difference.SASL, na.rm = TRUE) * 1.96)

line.lower.p10 <- mean(bland.altman$difference.SASL, na.rm = TRUE) - (sd(bland.altman$difference.SASL, na.rm = TRUE) * 1.96)

p10 <- ggplot(aes(x = bland.altman$mean.SASL, y = bland.altman$difference.SASL),

data = bland.altman) +

geom_point() +

xlim(0, 8) + ylim(-2, 2) +

labs(title = 'Short Axis Length, Systole (mm)', x = 'Average between examiners',

y = 'Difference between examiners', tag = 'l') +

theme(plot.title = element_text(size = 11)) + theme_bw() +

geom_hline(yintercept = 0, linetype = 1) +

geom_hline(yintercept = line.mean.p10, linetype = 2) +

geom_hline(yintercept = line.upper.p10, linetype = 2, color = 'blue') +

geom_hline(yintercept = line.lower.p10, linetype = 2, color = 'blue') +

annotate('text', x = 7.5, y = -0.2, size = 4, label = sprintf('%0.2f',line.mean.p10)) +

annotate('text', x = 7.5, y = 1.0, size = 4, color = 'blue', label = sprintf('%0.2f',line.upper.p10)) +

annotate('text', x = 7.5, y = -0.9, size = 4, color = 'blue', label = sprintf('%0.2f',line.lower.p10))

line.mean.p11 <- mean(bland.altman$difference.SADA, na.rm = TRUE)

line.upper.p11 <- mean(bland.altman$difference.SADA, na.rm = TRUE) + (sd(bland.altman$difference.SADA, na.rm = TRUE) * 1.96)

line.lower.p11 <- mean(bland.altman$difference.SADA, na.rm = TRUE) - (sd(bland.altman$difference.SADA, na.rm = TRUE) * 1.96)

p11 <- ggplot(aes(x = bland.altman$mean.SADA, y = bland.altman$difference.SADA),

data = bland.altman) +

geom_point() +

xlim(0, 25) + ylim(-4, 4) +

labs(title = 'Short Axis Area, Diastole' ~(mm^2), x = 'Average between examiners',

y = 'Difference between examiners', tag = 'g') +

theme(plot.title = element_text(size = 11)) + theme_bw() +

geom_hline(yintercept = 0, linetype = 1) +

geom_hline(yintercept = line.mean.p11, linetype = 2) +

geom_hline(yintercept = line.upper.p11, linetype = 2, color = 'blue') +

geom_hline(yintercept = line.lower.p11, linetype = 2, color = 'blue') +

annotate('text', x = 24, y = 0.8, size = 4, label = sprintf('%0.2f',line.mean.p11)) +

annotate('text', x = 24, y = 2.7, size = 4, color = 'blue', label = sprintf('%0.2f',line.upper.p11)) +

annotate('text', x = 24, y = -1.1, size = 4, color = 'blue', label = sprintf('%0.2f',line.lower.p11))

line.mean.p12 <- mean(bland.altman$difference.SASA, na.rm = TRUE)

line.upper.p12 <- mean(bland.altman$difference.SASA, na.rm = TRUE) + (sd(bland.altman$difference.SASA, na.rm = TRUE) * 1.96)

line.lower.p12 <- mean(bland.altman$difference.SASA, na.rm = TRUE) - (sd(bland.altman$difference.SASA, na.rm = TRUE) * 1.96)

p12 <- ggplot(aes(x = bland.altman$mean.SASA, y = bland.altman$difference.SASA),

data = bland.altman) +

geom_point() +

xlim(0, 20) + ylim(-5, 5) +

labs(title = 'Short Axis Area, Systole' ~(mm^2), x = 'Average between examiners',

y = 'Difference between examiners', tag = 'h') +

theme(plot.title = element_text(size = 11)) + theme_bw() +

geom_hline(yintercept = 0, linetype = 1) +

geom_hline(yintercept = line.mean.p12, linetype = 2) +

geom_hline(yintercept = line.upper.p12, linetype = 2, color = 'blue') +

geom_hline(yintercept = line.lower.p12, linetype = 2, color = 'blue') +

annotate('text', x = 19, y = 0.6, size = 4, label = sprintf('%0.2f',line.mean.p12)) +

annotate('text', x = 19, y = 2.7, size = 4, color = 'blue', label = sprintf('%0.2f',line.upper.p12)) +

annotate('text', x = 19, y = -1.6, size = 4, color = 'blue', label = sprintf('%0.2f',line.lower.p12))

jpeg(filename="Suppl Fig 2 Rev.jpeg", width=15, height=9, units='in', res=1200)

grid.arrange(p1, p2, p7, p8, p3, p4, p11, p12, p5, p6, p9, p10, ncol = 4)

dev.off()

##### FIGURE 3 #####

# Correlation for volumes, EF and FS

icc13 <- select(data, Examiner, Code, Bullet.EDV) %>% na.omit()

icc13 <- spread(icc13, Examiner, Bullet.EDV)

icc13.1 <- select(icc13, 'Examiner 1', 'Examiner 2')

cor13 <- ICC(icc13.1)

p13 <- ggplot(aes(x = examiner1$Bullet.EDV, y = examiner2$Bullet.EDV),

data = examiner1, examiner2) +

geom_point() +

xlim(0, 120) + ylim(0, 120) +

labs(title = 'End Diastolic Volume (ml)', x = 'Examiner 1', y = 'Examiner 2', tag = 'A') +

theme(plot.title = element_text(size = 11)) + theme_bw() +

annotate('text', x = 40, y = 107.5, size = 6, label = 'ICC = 0.97') +

geom_abline(color = 'red', size = 1, linetype = 2)

icc14 <- select(data, Examiner, Code, Bullet.ESV) %>% na.omit()

icc14 <- spread(icc14, Examiner, Bullet.ESV)

icc14.1 <- select(icc14, 'Examiner 1', 'Examiner 2')

cor14 <- ICC(icc14.1)

p14 <- ggplot(aes(x = examiner1$Bullet.ESV, y = examiner2$Bullet.ESV),

data = examiner1, examiner2) +

geom_point() +

xlim(0, 105) + ylim(0, 105) +

labs(title = 'End Systolic Volume (ml)', x = 'Examiner 1', y = 'Examiner 2', tag = 'B') +

theme(plot.title = element_text(size = 11)) + theme_bw() +

annotate('text', x = 30, y = 95, size = 6, label = 'ICC = 0.95') +

geom_abline(color = 'red', size = 1, linetype = 2)

icc15 <- select(data, Examiner, Code, Bullet.EF) %>% na.omit()

icc15 <- spread(icc15, Examiner, Bullet.EF)

icc15.1 <- select(icc15, 'Examiner 1', 'Examiner 2')

cor15 <- ICC(icc15.1)

p15 <- ggplot(aes(x = examiner1$Bullet.EF, y = examiner2$Bullet.EF),

data = examiner1, examiner2) +

geom_point() +

xlim(0, 100) + ylim(0, 100) +

labs(title = 'Ejection Fraction (%)', x = 'Examiner 1', y = 'Examiner 2', tag = 'E') +

theme(plot.title = element_text(size = 11)) + theme_bw() +

annotate('text', x = 30, y = 90, size = 6, label = 'ICC = 0.64') +

geom_abline(color = 'red', size = 1, linetype = 2)

icc16 <- select(data, Examiner, Code, twoD.FS) %>% na.omit()

icc16 <- spread(icc16, Examiner, twoD.FS)

icc16.1 <- select(icc16, 'Examiner 1', 'Examiner 2')

cor16 <- ICC(icc16.1)

p16 <- ggplot(aes(x = examiner1$twoD.FS, y = examiner2$twoD.FS),

data = examiner1, examiner2) +

geom_point() +

xlim(0, 1) + ylim(0, 1) +

labs(title = 'Fractional Shortening', x = 'Examiner 1', y = 'Examiner 2', tag = 'F') +

theme(plot.title = element_text(size = 11)) + theme_bw() +

annotate('text', x = 0.30, y = 0.9, size = 6, label = 'ICC = 0.50') +

geom_abline(color = 'red', size = 1, linetype = 2)

# Bland-Altman plots volumes, EF and FS

bland.altman <- mutate(examiner1, difference.EDV = examiner1$Bullet.EDV - examiner2$Bullet.EDV,

mean.EDV = (examiner1$Bullet.EDV + examiner2$Bullet.EDV) / 2,

difference.ESV = examiner1$Bullet.ESV - examiner2$Bullet.ESV,

mean.ESV = (examiner1$Bullet.ESV + examiner2$Bullet.ESV) / 2,

difference.EF = examiner1$Bullet.EF - examiner2$Bullet.EF,

mean.EF = (examiner1$Bullet.EF + examiner2$Bullet.EF) / 2,

difference.twoD.FS = examiner1$twoD.FS - examiner2$twoD.FS,

mean.twoD.FS = (examiner1$twoD.FS + examiner2$twoD.FS))

bland.altman <- mutate(bland.altman, percent.difference.EDV = difference.EDV/mean.EDV,

percent.difference.ESV = difference.ESV/mean.ESV,

percent.difference.EF = difference.EF/mean.EF,

percent.difference.twoD.FS = difference.twoD.FS/mean.twoD.FS)

mean(abs(bland.altman$percent.difference.EDV), na.rm = TRUE)

mean(abs(bland.altman$percent.difference.ESV), na.rm = TRUE)

mean(abs(bland.altman$percent.difference.EF), na.rm = TRUE)

mean(abs(bland.altman$percent.difference.twoD.FS), na.rm = TRUE)

line.mean.p17 <- mean(bland.altman$difference.EDV, na.rm = TRUE)

line.upper.p17 <- mean(bland.altman$difference.EDV, na.rm = TRUE) + (sd(bland.altman$difference.EDV, na.rm = TRUE) * 1.96)

line.lower.p17 <- mean(bland.altman$difference.EDV, na.rm = TRUE) - (sd(bland.altman$difference.EDV, na.rm = TRUE) * 1.96)

p17 <- ggplot(aes(x = bland.altman$mean.EDV, y = bland.altman$difference.EDV),

data = bland.altman) +

geom_point() +

xlim(0, 150) + ylim(-25, 25) +

labs(title = 'End Diastolic Volume (ml)', x = 'Average between examiners',

y = 'Difference between examiners', tag = 'C') +

theme(plot.title = element_text(size = 11)) + theme_bw() +

geom_hline(yintercept = 0, linetype = 1) +

geom_hline(yintercept = line.mean.p17, linetype = 2) +

geom_hline(yintercept = line.upper.p17, linetype = 2, color = 'blue') +

geom_hline(yintercept = line.lower.p17, linetype = 2, color = 'blue') +

annotate('text', x = 140, y = 3.5, size = 4, label = sprintf('%0.2f',line.mean.p17)) +

annotate('text', x = 140, y = 14, size = 4, color = 'blue', label = sprintf('%0.2f',line.upper.p17)) +

annotate('text', x = 140, y = -12, size = 4, color = 'blue', label = sprintf('%0.2f',line.lower.p17))

line.mean.p18 <- mean(bland.altman$difference.ESV, na.rm = TRUE)

line.upper.p18 <- mean(bland.altman$difference.ESV, na.rm = TRUE) + (sd(bland.altman$difference.ESV, na.rm = TRUE) * 1.96)

line.lower.p18 <- mean(bland.altman$difference.ESV, na.rm = TRUE) - (sd(bland.altman$difference.ESV, na.rm = TRUE) * 1.96)

p18 <- ggplot(aes(x = bland.altman$mean.ESV, y = bland.altman$difference.ESV),

data = bland.altman) +

geom_point() +

xlim(0, 100) + ylim(-20, 20) +

labs(title = 'End Systolic Volume (ml)', x = 'Average between examiners',

y = 'Difference between examiners', tag = 'D') +

theme(plot.title = element_text(size = 11)) + theme_bw() +

geom_hline(yintercept = 0, linetype = 1) +

geom_hline(yintercept = line.mean.p18, linetype = 2) +

geom_hline(yintercept = line.upper.p18, linetype = 2, color = 'blue') +

geom_hline(yintercept = line.lower.p18, linetype = 2, color = 'blue') +

annotate('text', x = 95, y = 2, size = 4, label = sprintf('%0.2f',line.mean.p18)) +

annotate('text', x = 95, y = 15, size = 4, color = 'blue', label = sprintf('%0.2f',line.upper.p18)) +

annotate('text', x = 95, y = -12, size = 4, color = 'blue', label = sprintf('%0.2f',line.lower.p18))

line.mean.p19 <- mean(bland.altman$difference.EF, na.rm = TRUE)

line.upper.p19 <- mean(bland.altman$difference.EF, na.rm = TRUE) + (sd(bland.altman$difference.EF, na.rm = TRUE) * 1.96)

line.lower.p19 <- mean(bland.altman$difference.EF, na.rm = TRUE) - (sd(bland.altman$difference.EF, na.rm = TRUE) * 1.96)

p19 <- ggplot(aes(x = bland.altman$mean.EF, y = bland.altman$difference.EF),

data = bland.altman) +

geom_point() +

xlim(0, 100) + ylim(-50, 50) +

labs(title = 'Ejection Fraction (%)', x = 'Average between examiners',

y = 'Difference between examiners', tag = 'G') +

theme(plot.title = element_text(size = 11)) + theme_bw() +

geom_hline(yintercept = 0, linetype = 1) +

geom_hline(yintercept = line.mean.p19, linetype = 2) +

geom_hline(yintercept = line.upper.p19, linetype = 2, color = 'blue') +

geom_hline(yintercept = line.lower.p19, linetype = 2, color = 'blue') +

annotate('text', x = 95, y = 7, size = 4, label = sprintf('%0.2f',line.mean.p19)) +

annotate('text', x = 95, y = 31, size = 4, color = 'blue', label = sprintf('%0.2f',line.upper.p19)) +

annotate('text', x = 95, y = -16, size = 4, color = 'blue', label = sprintf('%0.2f',line.lower.p19))

line.mean.p20 <- mean(bland.altman$difference.twoD.FS, na.rm = TRUE)

line.upper.p20 <- mean(bland.altman$difference.twoD.FS, na.rm = TRUE) + (sd(bland.altman$difference.twoD.FS, na.rm = TRUE) * 1.96)

line.lower.p20 <- mean(bland.altman$difference.twoD.FS, na.rm = TRUE) - (sd(bland.altman$difference.twoD.FS, na.rm = TRUE) * 1.96)

p20 <- ggplot(aes(x = bland.altman$mean.twoD.FS, y = bland.altman$difference.twoD.FS),

data = bland.altman) +

geom_point() +

xlim(0, 1) + ylim(-0.6, 0.6) +

labs(title = 'Fractional Shortening', x = 'Average between examiners',

y = 'Difference between examiners', tag = 'H') +

theme(plot.title = element_text(size = 11)) + theme_bw() +

geom_hline(yintercept = 0, linetype = 1) +

geom_hline(yintercept = line.mean.p20, linetype = 2) +

geom_hline(yintercept = line.upper.p20, linetype = 2, color = 'blue') +

geom_hline(yintercept = line.lower.p20, linetype = 2, color = 'blue') +

annotate('text', x = 0.95, y = 0.2, size = 4, label = sprintf('%0.2f',line.mean.p20)) +

annotate('text', x = 0.95, y = 0.4, size = 4, color = 'blue', label = sprintf('%0.2f',line.upper.p20)) +

annotate('text', x = 0.95, y = -0.30, size = 4, color = 'blue', label = sprintf('%0.2f',line.lower.p20))

jpeg(filename="Figure 3 Rev.jpeg", width=15, height=6, units='in', res=1200)

grid.arrange(p13, p14, p17, p18, p15, p16, p19, p20, ncol=4)

dev.off()

##### FIGURE 1 #####

# Volumes, EF and FS comparisons per time and infection status

p21 <- ggplot(aes(x = Timepoint, y = Average.EDV.bsa, color = Treatment), data = examiner1) + geom_boxplot(size = 1) +

#geom_point(position = position_dodge(width = 0.75)) +

ylim(0, 170) + labs(title = '', x = '', y = expression(EDV/BSA~(mL/m^2)), tag = 'A') + theme_bw(base_size = 14) +

scale_color_manual(values = c('grey0', 'grey50'), name = '', guide=FALSE) +

annotate('text', x = 1.81, y = 160, size = 4, label = '@') +

annotate('text', x = 2.195, y = 160, size = 4, label = '$')

p22 <- ggplot(aes(x = Timepoint, y = Average.ESV.bsa, color = Treatment), data = examiner1) + geom_boxplot(size = 1) +

#geom_point(position = position_dodge(width = 0.75)) +

ylim(0, 120) + labs(title = '', x = '', y = expression(ESV/BSA~(mL/m^2)), tag = 'B') + theme_bw(base_size = 14) +

scale_color_manual(values = c('grey0', 'grey50'), name = '', guide=FALSE) +

annotate('text', x = 1.81, y = 100, size = 4, label = '@') +

annotate('text', x = 2.19, y = 100, size = 4, label = '$')

p23 <- ggplot(aes(x = Timepoint, y = Average.EF, color = Treatment), data = examiner1) + geom_boxplot(size = 1) +

#geom_point(position = position_dodge(width = 0.75)) +

ylim(0, 100) + labs(title = '', x = '', y = 'Ejection Fraction (%)', tag = 'C') + theme_bw(base_size = 14) +

scale_color_manual(values = c('grey0', 'grey50'), name = '', guide=FALSE) +

annotate('text', x = 1.81, y = 80, size = 4, label = 'ns') +

annotate('text', x = 2.19, y = 80, size = 4, label = '$') +

annotate('text', x = 2.19, y = 95, size = 4, label = '&') + geom_hline(yintercept = 40, linetype = 2, color = 'red', size = 1)

p24 <- ggplot(aes(x = Timepoint, y = Average.twoD.FS, color = Treatment), data = examiner1) + geom_boxplot(size = 1) +

#geom_point(position = position_dodge(width = 0.75)) +

ylim(0, 1) + labs(title = '', x = '', y = 'Fractional Shortening', tag = 'D') + theme_bw(base_size = 14) +

scale_color_manual(values = c('grey0', 'grey50'), name = '', guide=FALSE) +

annotate('text', x = 1.81, y = 0.6, size = 4, label = 'ns') +

annotate('text', x = 2.19, y = 0.6, size = 4, label = '$') + geom_hline(yintercept = .2, linetype = 2, color = 'red', size = 1)

jpeg(filename="Figure 1 Rev.jpeg", width=9, height=6, units='in', res=1200)

grid.arrange(p21, p22, p23, p24, ncol=2)

dev.off()

# Two way ANOVA, Tukey's post-test and descriptive statistics for volumes, EF and FS

anovaEDV <- aov(Average.EDV.bsa ~ Treatment * Timepoint, data = examiner1)

Anova(anovaEDV, type = 3)

TukeyHSD(anovaEDV)

anovaESV <- aov(Average.ESV.bsa ~ Treatment * Timepoint, data = examiner1)

Anova(anovaESV, type = 3)

TukeyHSD(anovaESV)

anovaEF <- aov(Average.EF ~ Treatment * Timepoint, data = examiner1)

Anova(anovaEF, type = 3)

TukeyHSD(anovaEF)

anovaFS <- aov(Average.twoD.FS ~ Treatment * Timepoint, data = examiner1)

Anova(anovaFS, type = 3)

TukeyHSD(anovaFS)

stats.NI.BI <- filter(examiner1, Treatment == 'Non-infected', Timepoint == 'Before infection') %>%

select(Average.EDV.bsa, Average.ESV.bsa, Average.EF, Average.twoD.FS) %>% na.omit()

summary(stats.NI.BI)

stats.NI.AI <- filter(examiner1, Treatment == 'Non-infected', Timepoint == 'After infection') %>%

select(Average.EDV.bsa, Average.ESV.bsa, Average.EF, Average.twoD.FS)

summary(stats.NI.AI)

stats.I.BI <- filter(examiner1, Treatment == 'Infected', Timepoint == 'Before infection') %>%

select(Average.EDV.bsa, Average.ESV.bsa, Average.EF, Average.twoD.FS) %>% na.omit()

summary(stats.I.BI)

stats.I.AI <- filter(examiner1, Treatment == 'Infected', Timepoint == 'After infection') %>%

select(Average.EDV.bsa, Average.ESV.bsa, Average.EF, Average.twoD.FS) %>% na.omit()

summary(stats.I.AI)

##### FIGURE 2 #####

# Subsetting volumes by EF values below 40%

below.40 <- filter(examiner1, Timepoint == 'Post infection', Average.EF <= 40)

controls <- filter(examiner1, Timepoint == 'Post infection', Treatment == 'Non-infected')

data.40 <- rbind(below.40, controls)

p28 <- ggplot(aes(x = Treatment, y = Average.EDV.bsa), data = data.40) + geom_boxplot(size = 1) +

ylim(0, 170) + labs(title = '', x = '', y = expression(EDV/BSA~(mL/m^2)), tag = 'B') + theme_bw(base_size = 14) +

scale_color_manual(values = c('grey0', 'grey50'), name = '', guide=FALSE)

p29 <- ggplot(aes(x = Treatment, y = Average.ESV.bsa), data = data.40) + geom_boxplot(size = 1) +

ylim(0, 120) + labs(title = '', x = '', y = expression(ESV/BSA~(mL/m^2)), tag = 'C') + theme_bw(base_size = 14) +

scale_color_manual(values = c('grey0', 'grey50'), name = '', guide=FALSE) +

annotate('text', x = 2, y = 100, size = 6, label = 'p=0.0755')

p30 <- ggplot(aes(x = cut.off, y = delta.EF), data = examiner1) + geom_boxplot(size = 1) +

ylim(-50, 40) + labs(title = '', x = '', y = expression(Delta*'EF'), tag = 'A') + theme_bw(base_size = 14) +

scale_color_manual(values = c('grey0', 'grey50'), name = '', guide=FALSE) +

annotate('text', x = 2, y = 4.5, size = 5, label = '#')

jpeg(filename="Figure 2 Rev.jpeg", width=12, height=3, units='in', res=1200)

grid.arrange(p30, p28, p29, ncol=3)

dev.off()

# t tests and descriptive statistics "below 40% EF dogs"

t.test(data.40$Average.EDV.bsa~data.40$Treatment)

t.test(data.40$Average.ESV.bsa~data.40$Treatment)

t.test(examiner1$delta.EF~examiner1$cut.off)

stats.I.AI.below40 <- filter(below.40, Treatment == 'Infected', Timepoint == 'After infection') %>%

select(Average.twoD.FS)

summary(stats.I.AI.below40)

##### SUPPL FIGURE 3 #####

# ICC, mean difference and mean percent difference comparisons for echocardiography measurements

id <- c('LA Length, Diast', 'LA Length, Syst', 'SA Area, Diast', 'SA Area, Syst',

'SA Length, Diast', 'SA Length, Syst', 'End Diastolic Volume', 'End Systolic Volume',

'Ejection Fraction', 'Fractional Shortening')

iccbar <- c(0.90, 0.85, 0.97, 0.94, 0.89, 0.90, 0.97, 0.95, 0.64, 0.50)

meandifbar <- c(-0.12, -0.21, 0.45, 0.09, 0.13, 0.00, 0.91, -0.51, 3.15, 0.02)

percentdifbar <- c(6.16, 10.18, 11.87, 18.68, 9.20, 10.53, 12.71, 23.43, 18.45, 26.42)

databar <- data.frame(id, iccbar, meandifbar, percentdifbar)

databar$id <- ordered(databar$id, c('LA Length, Diast', 'LA Length, Syst', 'SA Area, Diast', 'SA Area, Syst',

'SA Length, Diast', 'SA Length, Syst', 'End Diastolic Volume', 'End Systolic Volume',

'Ejection Fraction', 'Fractional Shortening'))

p25 <- ggplot(aes(x = id, y = iccbar), data = databar) + geom_bar(stat = 'identity') +

ylim(0, 1) + labs(title = '', x = '', y = 'ICC', tag = 'a') + theme_bw(base_size = 14) +

theme(axis.text.x = element_text(angle = 45, vjust = 1, hjust=1))

p26 <- ggplot(aes(x = id, y = meandifbar), data = databar) + geom_bar(stat = 'identity') +

ylim(-1, 5) + labs(title = '', x = '', y = 'Mean Diference', tag = 'b') + theme_bw(base_size = 14) +

theme(axis.text.x = element_text(angle = 45, vjust = 1, hjust=1))

p27 <- ggplot(aes(x = id, y = percentdifbar), data = databar) + geom_bar(stat = 'identity') +

ylim(0, 30) + labs(title = '', x = '', y = 'Mean Percent Difference', tag = 'c') + theme_bw(base_size = 14) +

theme(axis.text.x = element_text(angle = 45, vjust = 1, hjust=1))

jpeg(filename="Suppl Fig 3 Rev.jpeg", width=12, height=6, units='in', res=1200)

grid.arrange(p25, p26, p27, ncol=3)

dev.off()

##################################

#END#

##################################
